# Penalized regression approaches to testing for quantitative trait-rare variant association

**DOI:** 10.3389/fgene.2014.00121

**Published:** 2014-05-13

**Authors:** Sunkyung Kim, Wei Pan, Xiaotong Shen

**Affiliations:** ^1^Division of Biostatistics, School of Public Health, University of MinnesotaMinneapolis, MN, USA; ^2^School of Statistics, University of MinnesotaMinneapolis, MN, USA

**Keywords:** GWAS, SSU test, SSUw test, Sum test, TLP

## Abstract

In statistical data analysis, penalized regression is considered an attractive approach for its ability of simultaneous variable selection and parameter estimation. Although penalized regression methods have shown many advantages in variable selection and outcome prediction over other approaches for high-dimensional data, there is a relative paucity of the literature on their applications to hypothesis testing, e.g., in genetic association analysis. In this study, we apply several new penalized regression methods with a novel penalty, called Truncated *L_1_*-penalty (TLP) (Shen et al., 2012), for either variable selection, or both variable selection and parameter grouping, in a data-adaptive way to test for association between a quantitative trait and a group of rare variants. The performance of the new methods are compared with some existing tests, including some recently proposed global tests and penalized regression-based methods, via simulations and an application to the real sequence data of the Genetic Analysis Workshop 17 (GAW17). Although our proposed penalized methods can improve over some existing penalized methods, often they do not outperform some existing global association tests. Some possible problems with utilizing penalized regression methods in genetic hypothesis testing are discussed. Given the capability of penalized regression in selecting causal variants and its sometimes promising performance, further studies are warranted.

## 1. Introduction

Genome-wide association studies (GWAS) have uncovered many common variants (CVs) associated with complex diseases, but the proportion of variance explained by the identified CVs is often low (Maher, 2008). With the recent advance of sequencing technologies, analysis of rare variants (RVs) has become a feasible alternative. Recent studies have demonstrated that some RVs are associated with complex disease. For example, Kotowski et al. (2006) found that multiple RVs in gene PCSK9 are associated with plasma levels of low-density lipoprotein cholesterol.

In this study, we propose applying some new penalized regression methods to test for association between a quantitative trait and multiple RVs. Differing from the usual application of penalized regression methods to variable selection or risk prediction for high-dimensional data (Kooperberg et al., 2010; Austin et al., 2013), here we focus on their application to hypothesis testing on a quantitative trait in a relatively low-dimensional setting. In such a setting, one commonly used statistical test is the *F*-test in linear regression. For example, in simple regression, a trait *Y* is regressed on each of multiple variants sequentially. However, because of the extremely low minor allele frequency (MAF) of a RV, a test to detect the association between a trait and a single RV might be low powered. Also, this approach may be too conservative due to a stringent control for multiple testing, e.g., by the Bonferroni correction to control the family-wise error rate. In addition, ultimately, complex diseases are expected to be affected by a combination of multiple genetic variants. Thus an analysis in which a group of variants are tested simultaneously for their joint effects on the trait may be more powerful. In multiple regression, to assess any association between a trait and *k* RVs, all *k* RVs are added to a regression model. However, as *k* increases, the statistical power might decrease due to the cost of large degrees of freedom (DF), *k*. To avoid the large DF and to aggregate information across multiple RVs, one common strategy is to pool or collapse multiple RVs in a region or gene (Li and Leal, 2008; Madsen and Browning, 2009). One such attempt is the Sum test (Pan, 2009), which was developed to utilize joint effects of multiple variants while reducing the DF. With only 1 DF, the Sum test enhances power under some scenarios (Chapman and Whittaker, 2008; Pan, 2009). However, it is noted that the performance of the Sum test depends on the directions of the variants' associations with a trait. Thus, in an extreme case where a half of the variants are positively associated with the trait and the other half are negatively associated with similar effect sizes, the positive and negative effects may cancel out, leading to the poor performance of the Sum test and other burden tests (Han and Pan, 2010; Li et al., 2010). In addition, in the Sum test or other pooling-based burden tests, combining or collapsing all variants into just one group ignores the variants' possibly varying effect sizes, and thus may not work well in those situations. In particular, the Sum test and many burden tests perform poorly if many null (i.e., non-associated) RVs are present (Basu and Pan, 2011). Consequently, the Sum test and other pooled association tests might be low powered.

On the other hand, to deal with high-dimensional genetic and genomic data, penalized regression methods have received much attention, especially those based on the Lasso penalty (Tibshirani, 1996; Kooperberg et al., 2010). Penalized regression has been considered attractive for its potential of simultaneous variable selection and parameter estimation. In particular, several authors have studied the performance of penalized regression in genetic association analysis (Guo and Lin, 2009; Tzeng and Bondell, 2010; Zhou et al., 2011). However, the penalties used therein are typically based on the Lasso, which is known to give biased parameter estimates and possibly inconsistent variable selection. In contrast, one of the very recently developed state-of-the-art penalties, the truncated *L*_1_-penalty (TLP) (Shen et al., 2012), overcomes the above shortcomings of Lasso. The TLP approximates the *L*_0_-loss and reduces the bias of a parameter estimate from the popular Lasso or *L*_1_-penalty. To investigate whether an application of TLP would boost statistical power in genetic association testing, in this study we apply the TLP for variable selection, denoted TLP-S, and for both variable selection and parameter grouping (Zhu et al., 2013), denoted TLP-SG, in a data-adaptive way, to select and group variants to reduce the DF as in the Sum test, while reducing the downward bias of the parameter estimates based on an *L*_1_-type penalty. We compare the TLP-S and TLP-SG to the Lasso and graph-fused Lasso (gflasso) (Kim and Xing, 2009). The gflasso also pursues parameter grouping with an *L*_1_-penalty. Specifically, the gflasso shrinks two variants' effect sizes toward each other by penalizing their difference |β_*j*_ − *r*(*j, j*′)β_*j*′_|, where either *r*(*j, j*′) = 1 (called gflasso_*r* = 1_) or *r*(*j, j*′) is the sign of the correlation between the two variants *j* and *j*′ (called flasso_*r = cor*_). There are two main differences between our proposed TLP-SG and gflasso. First, TLP-SG shrinks the absolute values of the two parameters toward each other by penalizing $\left|\left|\beta_{j}|-|\beta_{j^{\prime }}\right|\right|$. In this way, it desirably allows two variants to have similar effect sizes but opposite association directions. However, such a penalty is non-convex and thus computationally more challenging. Second, by the use of TLP-based grouping (see details later), TLP-SG shrinks |β_*j*_| and |β_*j*′_| toward each other only if their difference is relatively small (as compared to a tuning parameter to be decided), thus, for example, avoiding severely biasing the estimate of the effect size of an associated variant toward 0 by shrinking it toward the null effect of a null variant. We note that, although penalized regression methods have been widely used and studied, their applications to the current context with RVs are much more limited; in particular, we are not aware of any applications of TLP-S, TLP-SG and gflasso to association testing of RVs.

This paper is organized in four sections. Section 2 provides a brief review of some existing association tests to be compared, and then introduces our proposed TLP-based tests. In section 3, we compare the performance of the methods with simulated data and with an application to the Genetic Analysis Workshop 17 (GAW17) sequence data (Almasy et al., 2011). Finally, the Discussion section summarizes the results, and suggests some potential problems for future study.

## 2. Methods

### 2.1. Some existing association tests

We briefly review some existing global tests based on the ordinary least squares (OLS) estimates. Given *n* independent observations (*Y_i_, X_i_*), *i* = 1, …, *n*, with *Y*_*i*_ as a quantitative trait and a vector *X*_*i*_ = (*X*_*i*1_, …, *X_ik_*) as genotypes of *k* variants for subject *i*, we would like to test for any possible association between the trait and genotypes. We use the dosage coding for *X*_*ij*_: *X*_*ij*_ = 0, 1, or 2, representing the count number of one of the two alleles present in variant *j* of subject *i*. A multi-locus association analysis is based on fitting a linear model,

(1) $$Y_{i}=\beta_{0}+\sum_{j=1}^{k}X_{ij}\beta_{j}+\epsilon_{i}$$

where the errors ϵ_*i*_ are assumed to be independently drawn from *N*(0, σ^2^), a Normal distribution with mean 0 and variance σ^2^. A global test of any possible association between the trait and *k* variants can be formulated as testing on the multiple parameters β_*j*_s for *j* = 1, …, *k* with null hypothesis *H*_0_: β = (β_1_, …, β_*k*_)′ = 0 by an *F*-test, which is based on the OLS estimates that minimize the residual sum of squares. A potential problem with the test is the power loss due to the large variance of $\hat{\beta }$_*j*_ since the MAFs of RVs are small.

We also apply other four association tests: the Score, the sum of squared score (SSU), its weighted version SSUw (Pan, 2009), and the univariate minP (UminP) tests. The Score test is popular in general statistics while the UminP test is most widely used for CVs in GWAS; on the other hand, Basu and Pan (2011) showed that the SSU and SSUw tests were powerful in RV association testing for case-control studies. Here, as a secondary contribution, we extend the SSU and SSUw tests to the case with a quantitative trait. All the four tests are based on the score vector *U* and its covariance matrix *V* under *H*_0_:

$$\begin{array}{l} U=\sum_{i=1}^{n}\left(Y_{i}-\bar{Y}\right)X_{i},\\ V=Cov(U)={\hat{\sigma }}_{o}^{2}\sum_{i=1}^{n}\left(X_{i}-\bar{X}\right){\left(X_{i}-\bar{X}\right)}^{T}, \end{array}$$

where $\bar{Y}=\sum_{i=1}^{n}Y_{i}/n,\bar{X}=\sum_{i=1}^{n}X_{i}/n$, and ${\hat{\sigma }}_{o}^{2}=\sum_{i=1}^{n}{\left(Y_{i}-\bar{Y}\right)}^{2}/(n-1)$ is the estimate of σ^2^ under *H*_0_. The corresponding four test statistics are

$$\begin{array}{l} T_{Score}=U^{T}V^{-1}U,\\ \;T_{SSU}=U^{T}U,\\ \;T_{SSUw}=U^{T}V_{d}^{-1}U\text{ with }V_{d}=Diag(V),\\ T_{UminP}=\overset{k}{\underset{j=1}{\max}}U_{j}^{2}/v_{j}, \end{array}$$

where *U*_*j*_ is the *j*th element of *U* and *v*_*j*_ is the (*j, j*)th diagonal element of *V*. Under *H*_0_, asymptotically *T*_*Score*_ has a χ^2^_*k*_ distribution, each of *T*_*SSU*_ and *T*_*SSUw*_ has a mixture of chi-squared distributions (Pan, 2009), and the *p*-value of *T*_*UminP*_ can be numerically obtained (Conneely and Boehnke, 2007).

Next, we extend the Sum test (Pan, 2009) and its modified version, a data-adaptive Sum (aSum) test (Han and Pan, 2010), to the case with a quantitative trait. The Sum test was originated to model multiple variants jointly while inducing a minimum number of DF: while including all the variants in the linear model, it assumes that the variants all have the same effect size (and direction), β_*c*_, as in the following model:

(2) $$Y_{i}=\beta_{c,0}+\sum_{j=1}^{k}X_{ij}\beta_{c}+\epsilon_{i}$$

Fitting (Equation 2) is equivalent to conducting a simple regression of *Y* on a new covariate, the sum of the genotypes over the multiple variants. To address the question of whether any association between the disease and the variants exists, one simply needs to test *H*_0_ : β_*c*_ = 0, without the need for multiple testing adjustment. The main advantage of the Sum test is that, because it tests on only one parameter β_*c*_, there will be no power loss due to the large DF. The common association parameter β_*c*_ is a weighted average of the individual β_*M*, 1_, …, β_*M, k*_ in the marginal models *Y*_*i*_ = β_*M*, 0_ + *X_ij_*β_*M, j*_ + ϵ_*ij*_ for *j* = 1, …, *k* (Pan, 2009). On the other hand, the main problem of the Sum test is its dependence on the signs of β_*M,j*_s or on the coding of each variant (i.e., which allele is chosen as the reference category). If the signs are not the same, the test may have a quite small $\hat{\beta }$_*c*_ and thus low power. To overcome the limitation of the Sum test, Han and Pan (2010) proposed the aSum test for a case-control study design, which can be equally applied to quantitative traits as the following. (1) For each variant *j*, flip its coding to X^*^_.*j*_ = 2 − *X_.j_* if $\hat{\beta }$_*M,j*_ < 0 and its *p*-value *p*_*M,j*_ ≤ α_0_ in the marginal model; otherwise use the same coding X^*^_.*j*_ = *X_.j_*. (2) Fit the model (Equation 2) with the new coding X^*^. To test *H*_0_ in the aSum test, we use a permutation-based log-likelihood ratio test (LRT), which is asymptotically equivalent to the score test. For the choice of α_0_, we use the same value as recommended by Han and Pan (2010), 0.1, to prevent reduced power when a too small or too large α_0_ is used.

While the *F*-test is based on OLS estimates, in next section we apply some penalized regression methods, the Lasso, gflasso and a recently developed TLP for either only variable selection (TLP-S) or both variable selection and parameter grouping (TLP-SG). In short, both the Lasso and TLP-S consider only variable selection, while the gflasso and TLP-SG pursue parameter grouping along with variable selection to improve power by striking a better balance between goodness-of-fit and reduced DF in the joint model (Equation 1).

### 2.2. Penalized regression based tests

#### 2.2.1. Parameter estimation from penalized regression

**Figure 1:**
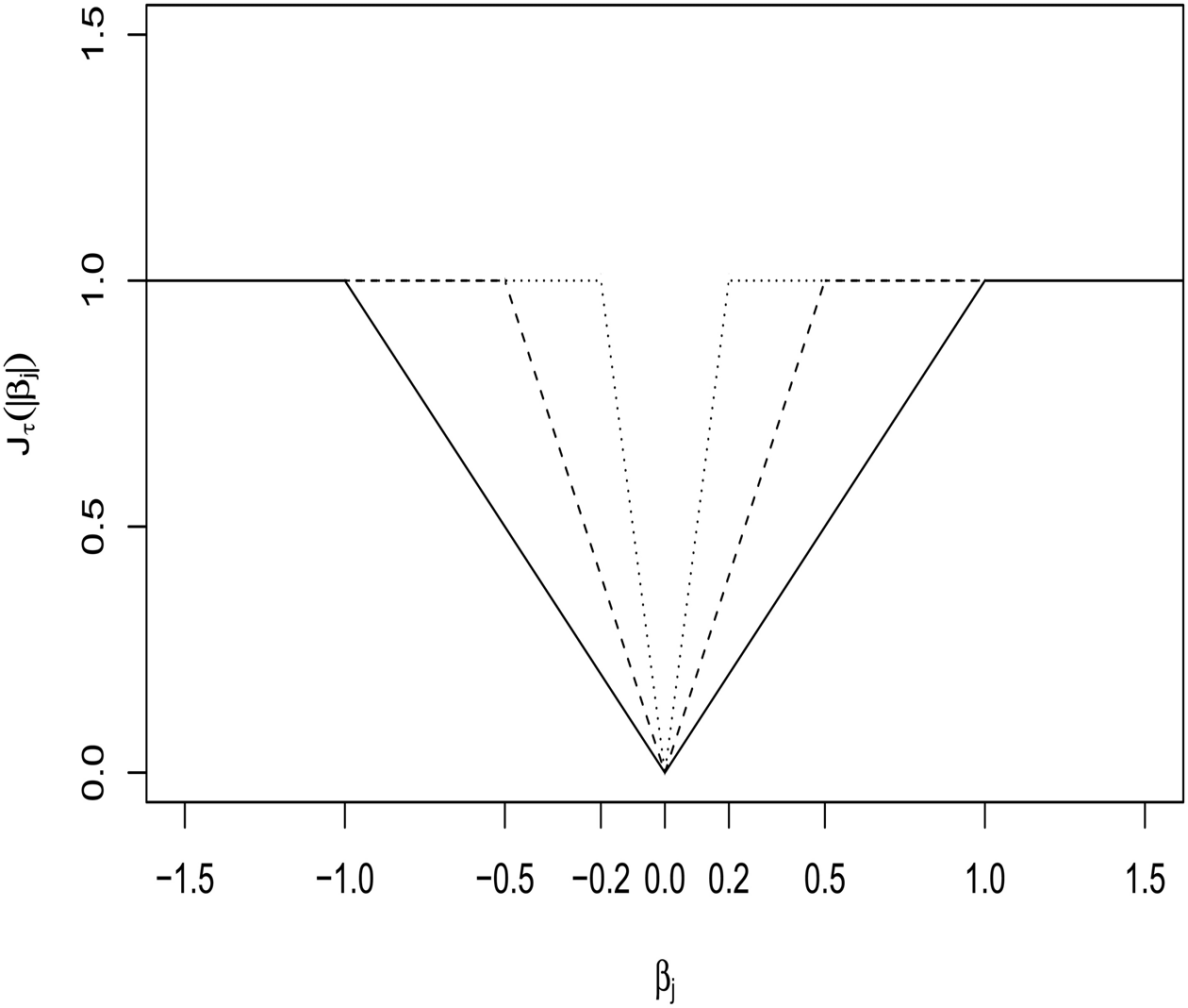
**Truncated *L*_1_-penalty (TLP) function *J*_τ_(|β_*j*_|) with τ = 0.2, 0.5, and 1 (as solid, dashed and dotted lines, respectively)**.

Given a vector of traits *Y* = (*Y*_1_, …, *Y_n_*)′ and a design matrix for *k* variants *X* = (*X*_·1_, …, *X*_·*k*_), the Lasso estimate of β is obtained from the penalized least squares function:

(3) $$\hat{\beta }=\underset{\beta }{\mathrm{argmin}}\frac{1}{2}\|Y-X\beta {\|}^{2}+\lambda \sum_{j=1}^{k}|\beta_{j}|,$$

where a large λ automatically yields some components of $\hat{\beta }$ as 0, realizing variable selection. While Lasso does effective variable selection, its estimates are always biased. To overcome the issue, Shen et al. (2012) proposed a truncated Lasso(*L*_1_)-penalty (TLP) $J_{\tau }(|x|)=min(\frac{\left|x\right|}{\tau },1)$, which, as τ → 0^+^, tends to the *L*_0_-loss, *I*(|*x*| ≠ 0). The degree of approximation by TLP is controlled by a tuning parameter, τ. See Figure 1 for a display over the different values of τ. Then the TLP-estimate $\hat{\beta }$ is obtained from

(4) $$\hat{\beta }=\underset{\beta }{\mathrm{argmin}}\frac{1}{2}\|Y-X\beta {\|}^{2}+\lambda_{1}\sum_{j=1}^{k}J_{\tau }(|\beta_{j}|),$$

and we denote (Equation 4) as TLP-S. The most interesting feature of the TLP is that only smaller |β_*j*_|'s less than a threshold τ are penalized, hence realizing variable selection (if some are shrunken to 0) while avoiding penalizing larger |β_*j*_|'s and thus leading to their almost unbiased estimates.

While both the Lasso and TLP-S consider only variable selection, an alternative way to reduce model complexity is grouping pursuit (Shen and Huang, 2010). To investigate the grouping effects on a test's power, we apply two recent penalized grouping methods, gflasso and TLP-SG. The β estimate from gflasso is based on the following objective function:

(5) $$\begin{array}{l} \hat{\beta }=\underset{\beta }{\mathrm{argmin}}\frac{1}{2}\|Y-X\beta {\|}^{2}+\lambda_{1}\sum_{j=1}^{k}|\beta_{j}|\\ \;+\lambda_{2}\sum_{j<j^{\prime }}|\beta_{j}-r(j,j^{\prime })\beta_{j^{\prime }}|, \end{array}$$

where the first penalty is used for variable selection and the second is to encourage parameter grouping. *r*(*j, j*′) is the sign of the correlation between two variants *X*_·*j*_ and *X*_·*j*′_, which is used to approximate the target |β_*j*_| ≈ |β_*j*′_|; this method is denoted gflasso_*r = cor*_. On the other hand, if *r*(*j, j*′) = 1 is used, the penalty targets β_*j*_ ≈ β_*j*′_.

The TLP-SG estimate of β comes from

(6) $$\begin{array}{l} \hat{\beta }=\underset{\beta }{\mathrm{argmin}}\frac{1}{2}\|Y-X\beta {\|}^{2}+\lambda_{1}\sum_{j=1}^{k}J_{\tau }(|\beta_{j}|)\\ \;+\lambda_{2}\sum_{j^{\prime }<j}J_{\tau }(||\beta_{j}|-|\beta_{j^{\prime }}||), \end{array}$$

where the first penalty is for variable selection while the second shrinks the difference of |β_*j*_|'s if a difference is within the upper bound τ. The number of the groups of equal parameter estimates is a decreasing function of λ_2_. Thus, the tuning parameters (λ_1_, λ_2_, τ) are selected to balance between the model complexity and model goodness-of-fit, which presumably may contribute to enhanced power. As a comparison, in the Sum test all parameters (or variants) are forced to belong to the same single group even if the variants' associations with the trait are quite different both in effect sizes and directions; the TLP-SG method attempts to conduct a more precise grouping over all variants in a data-adaptive way.

To compute β in Lasso, gflasso, TLP-S and TLP-SG, we used the Feature Grouping and Selection Over an Undirected Graph (FGSG) package of Yang et al. (2012), which is a C library with interface to MATLAB and is quite fast to run. Its computing efficiency allowed us to estimate separate tuning parameters for each permuted dataset to control the type I error as explained in the next section.

#### 2.2.2. Hypothesis testing

To test the null hypothesis *H*_0_ : β = 0 in Equation (1), we conduct a permutation-based test, in which the *p*-value is calculated by comparing a test statistic *T* applied to the original dataset to the ones *T*^(*b*)^_0_ applied to the *B* permuted datasets for *b* = 1, …, *B*. We use permutation to control the Type I error since the null distribution of a test statistic based on a penalized regression estimate is in general difficult to obtain. The permutation-based testing procedure follows:

1. Step 1. With the original data {(*Y*_*i*_, *X*_*i*_)}, we solve a penalized regression problem to obtain $\hat{\beta }$ in Equation (1).
2. Step 2. Calculate a test statistic *T* = *T*($\hat{\beta }$).
3. Step 3. By repeatedly permuting the observed *Y* of the original data, we obtain *B* sets of permuted data {(*Y^(b)^*_*i*_, *X*_*i*_)} for *b* = 1, …, *B*. For each permuted data set, {(*Y^(b)^*_*i*_, *X*_*i*_)}, we repeat the Steps 1 and 2, obtaining the null statistics *T*^*(b)*^_0_.
4. Step 4. The final *p*-value is $\sum_{b=1}^{B}I\left(T<T_{0}^{\left(b\right)}\right)/B$.

We apply each of several test statistics in Step 2. First, across all penalized methods, we use a 1-df *F*-statistic (1-df) to test the association between *Y* and *X*$\hat{\beta }$, where $\hat{\beta }$ is the penalized estimate of β in Step 1. Specifically, we fit a linear model

$$Y_{i}=\alpha_{0}+\left(X_{i}\hat{\beta }\right)\alpha +\epsilon_{i},$$

and test *H*′_0_ : α = 0. This 1-df test uses variable selection and possibly parameter grouping result from the corresponding penalized method, while allowing testing with only 1 DF. Second, for TLP-SG, we also apply the corresponding SSU and SSUw tests, where the test statistics *T*_*SSU*_ and *T*_*SSUw*_ are both based on the selected variables from the corresponding penalized estimates. Specifically,

$$\begin{array}{l} \;T_{SSU}=U^{{*}^{\prime }}U^{*},\\ T_{SSUw}=U^{{*}^{\prime }}{\left(V_{d}^{*}\right)}^{-1}U^{*}\text{ with }V_{d}^{*}=Diag(V^{*}), \end{array}$$

where U^*^ is a sub-component vector of the score vector *U* corresponding to |$\hat{\beta }$_*j*_| ≠ 0, and |$\hat{\beta }$_*j*_| > 0.001 is considered as non-zero. Similarly, V^*^ is the corresponding sub-matrix of the covariance matrix *V*. Note that the grouping information is not used.

#### 2.2.3. Selection of tuning parameters

To select the suitable tuning parameters, we apply a grid-search with Akaike's information criterion (AIC) (Akaike, 1974):

AIC=−2logL+2p,

where log *L* = (−*n*log($\hat{\sigma }$^2^) − *n* − *p* − 1)/2 is the log-likelihood with the penalized estimate plugged-into model (Equation 1), and ${\hat{\sigma }}^{2}=\sum_{i=1}^{n}{\left(Y_{i}-\beta_{0}-X_{i}\hat{\beta }\right)}^{2}/(n-p-1)$. The effective number of the parameters, *p*, in AIC is computed as the number of non-zero |$\hat{\beta }$_*j*_|'s for Lasso and TLP-S, as the number of non-zero unique $\hat{\beta }$_*j*_'s for gflasso_*r* = 1_, and as the number of non-zero unique |$\hat{\beta }$_*j*_|'s for gflasso_*r = cor*_ and TLP-SG, respectively. For λ in Lasso, the one resulting in the smallest AIC out of 50 equally spaced points in [0.001,10] is selected. Similarly, the values of each of λ_1_, λ_2_ and τ in other methods are searched over five equally spaced grid points of [0.001, 1], [0.001, 0.5], and [0.001, 0.5], respectively. For each permuted dataset {(*Y*^*(b)*^_*i*_, *X*_*i*_)} for *b* = 1, …, *B*, we also estimate its own (λ^*(b)*^_1_, λ^*(b)*^_2_, τ^*(b)*^) to properly control the type I error.

## 3. Results

### 3.1. Simulations

We consider two simulation schemes. In the first scheme, we generate only RVs with a total of 200 replicates and *n* = 400 in each replicate. The permutation size is set as *B* = 100. For each replicate, to generate *k* variants including six causal ones in linkage disequilibrium (LD), as in Wang and Elston (2007), two latent vectors from multivariate normal distribution MVN(0,*R*) are simulated, where *R* has a first order auto regressive (AR1) structure; the association between any two elements of the latent vector decreases by ρ = 0.8 times as 1 lag increases. Then, the vector is dichotomized to yield a haplotype with the minor allele frequency (MAF) of each variant randomly chosen between 0.005 and 0.01. The genotype data *X*_*i*_ = (*X*_*i*1_, …, *X_ik_*)′ for sample *i* is obtained by adding two haplotypes together. Finally, *Y*_*i*_ is generated from the randomly located six causal variants with σ^2^ = 2 in model (Equation 1), where the intercept β_0_ is set as 0.3 throughout the simulations. The considered three cases are:

$$\begin{array}{l} \text{Case 1: }\beta =\left(\underset{6}{\underset{︸}{0.9,0.9,0.9,0.9,0.9,0.9}},\underset{k-6}{\underset{︸}{0,\ldots ,0}}\right)\prime \\ \text{Case 2: }\beta =\left(\underset{6}{\underset{︸}{1.2,1.2,1.2,-1.2,-1.2,-1.2}},\underset{k-6}{\underset{︸}{0,\ldots ,0}}\right)\prime \\ \text{Case 3: }\beta =\left(\underset{6}{\underset{︸}{1.4,1.3,-1.2,1.2,-1.3,1.4}},\underset{k-6}{\underset{︸}{0,\ldots ,0}}\right)\prime . \end{array}$$

In each case, we vary the number of non-causal RVs *k*-6 from 0 to 24 so that the total number of RVs, *k*, ranges from 6 to 30. The Type I error is computed from the *Y* under *H*_0_ : β = (0, …, 0)′.

In the second scheme, multiple RVs and two CVs are generated to mimic the GAW17 data we use later. The frequency of one allele for each CV is randomly distributed between 0.2 and 0.7, and CVs may or may not be chosen as a causal variant in each replicate. When a CV is randomly selected as a causal variant, its effect size β_*j*_ is scaled down to β_*j*_/10 in the following cases to prevent its dominating association with the outcome. The considered three cases for mixed RVs and CVs are:

$$\begin{array}{l} \text{Case 1: }\beta =\left(\underset{6}{\underset{︸}{1,1,1,1,1,1}},\underset{k-6}{\underset{︸}{0,\ldots ,0}}\right)\prime \\ \text{Case 2: }\beta =\left(\underset{6}{\underset{︸}{1.5,1.5,1.5,-1.5,-1.5,-1.5}},\underset{k-6}{\underset{︸}{0,\ldots ,0}}\right)\prime \\ \text{Case 3: }\beta =\left(\underset{6}{\underset{︸}{1.1,1.3,-1.2,1.2,-1.3,1.1}},\underset{k-6}{\underset{︸}{0,\ldots ,0}}\right)\prime , \end{array}$$

**Figure 2:**
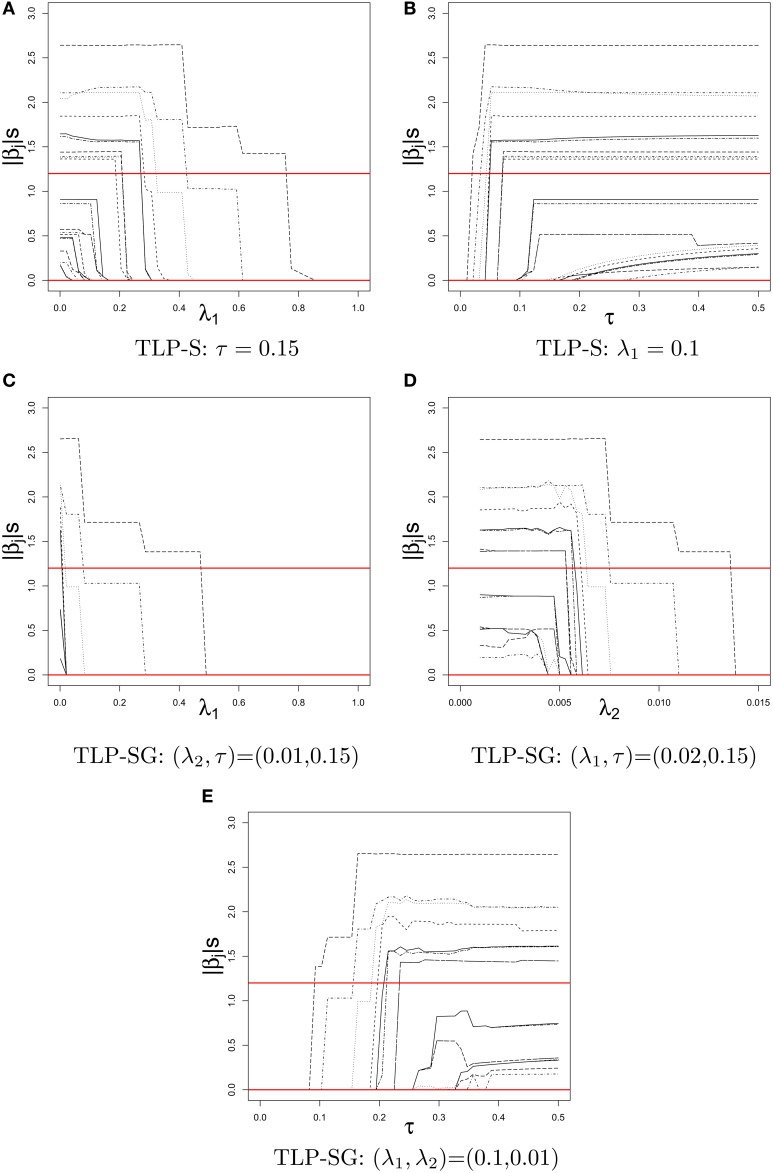
**Solution paths of |$\hat{\beta }$_*j*_|'s in a simulated dataset of Case 2 with *k* = 22 RVs for TLP-S and TLP-SG over the values of a tuning parameter given other(s)**. The true values of |β_*j*_|'s at 1.2 and 0 are given by two horizontal lines. **(A)** TLP-S: τ = 0.15. **(B)** TLP-S: λ_1_ = 0.1. **(C)** TLP-SG: (λ_2_, τ) = (0.01, 0.15). **(D)** TLP-SG: (λ_1_, τ) = (0.02, 0.15). **(E)** TLP-SG: (λ_1_, λ_2_) = (0.1, 0.01).

Figure 2 displays the TLP-S and TLP-SG solution paths of |$\hat{\beta }$_*j*_| over a tuning parameter given other(s), where two horizontal lines at 1.2 and 0 give the true parameter values for Case 2 set-up with only RVs. In contrast to piece-wise linear solution paths of the Lasso estimates, the TLP solution paths are like step functions as expected from an *L*_0_-penalty (i.e., best subset selection).

Table 1 presents the simulation results for the RVs only set-ups. The Type I error rates seem to be properly controlled under the null for all cases, though there are some slightly inflated numbers, possibly due to the relatively small number of replicates and/or permutation numbers. Under the alternative hypothesis, in Case 1 where the causal associations are all in the same direction, the Sum or aSum test beats other methods. Within the class of penalized regression methods, TLP-SG with the SSU or SSUw test statistics is most powerful; in particular, TLP-SG with the SSUw statistic performs better than the *F*-test regardless of the number of non-causal RVs included. There seems to be no gain with grouping in TLP-SG as compared to no grouping in TLP-S, and the 1df-test of TLP-SG works better than gflasso_*r = cor*_ unless the number of non-causal RVs is large at 24. Overall, penalized regression methods do not significantly outperform the power over the Sum and aSum tests. In Cases 2 and 3, where the causal effect directions are mixed, the Sum test works poorly as expected, while the aSum test has higher power. Overall, either the SSU or SSUw test is the winner. In particular, the TLP-S- and TLP-SG-based tests do not significantly improve over the SSU and SSUw tests, though they may perform better than those based on the Lasso and gflasso. Again, a comparison between TLP-S and TLP-SG reveals that parameter grouping does not seem to contribute much to increased power.

**Table 1:** **Empirical Type I error and Power at the nominal level α = 0.05 based on 200 replicates for the RVs only set-ups with six causal RVs and a varying number of non-causal RVs**.

**Model fitting**	**Test statistics**	**# of non-causal RVs**				**# of non-causal RVs**			
		**0**	**8**	**16**	**24**	**0**	**8**	**16**	**24**
		**Null**				**Case 1**			
OLS	*F*-test	0.030	0.080	0.040	0.060	0.715	0.480	0.340	0.260
OLS	Score	0.030	0.080	0.035	0.055	0.710	0.470	0.320	0.245
OLS	SSU	0.030	0.060	0.045	0.045	0.830	0.660	0.510	0.405
OLS	SSUw	0.035	0.080	0.055	0.060	0.810	0.625	0.500	0.380
OLS	UminP	0.045	0.070	0.050	0.035	0.675	0.445	0.360	0.310
OLS	Sum	0.055	0.075	0.040	0.075	**0.915**	0.685	0.525	0.460
OLS	aSum	0.035	0.065	0.035	0.060	0.910	**0.715**	**0.575**	**0.520**
Lasso	1df	0.055	0.075	0.050	0.080	0.710	0.415	0.325	0.270
gflasso_*r = cor*_	1df	0.035	0.080	0.050	0.090	0.690	0.415	0.240	0.295
gflasso_*r* = 1_	1df	0.035	0.070	0.050	0.075	0.685	0.375	0.225	0.275
TLP-S	1df	0.050	0.085	0.050	0.075	0.720	0.450	0.305	0.255
TLP-SG	1df	0.055	0.085	0.055	0.070	0.700	0.450	0.290	0.250
TLP-SG	SSU	0.055	0.080	0.040	0.060	0.700	0.520	0.440	0.390
TLP-SG	SSUw	0.040	0.075	0.045	0.070	0.790	0.500	0.365	0.320
		**Case 2**				**Case 3**			
OLS	*F*-test	**0.635**	0.515	0.440	0.455	0.745	0.640	0.550	0.490
OLS	Score	0.625	0.500	0.425	0.395	0.745	0.635	0.525	0.470
OLS	SSU	0.590	0.530	**0.505**	0.445	0.710	0.645	**0.595**	**0.555**
OLS	SSUw	0.570	0.505	0.475	0.445	0.715	**0.660**	0.570	0.525
OLS	UminP	0.450	0.410	0.400	0.310	0.665	0.595	0.425	0.425
OLS	Sum	0.145	0.125	0.145	0.100	0.485	0.310	0.260	0.215
OLS	aSum	0.450	0.430	0.355	0.340	0.665	0.590	0.535	0.500
Lasso	1df	0.615	0.465	0.405	0.390	**0.765**	0.585	0.465	0.435
gflasso_*r = cor*_	1df	0.620	0.530	0.435	**0.480**	**0.765**	0.600	0.480	0.520
gflasso_*r* = 1_	1df	0.615	**0.535**	0.435	0.425	0.750	0.585	0.475	0.495
TLP-S	1df	0.615	0.505	0.455	0.425	0.760	0.630	0.530	0.475
TLP-SG	1df	0.615	0.485	0.445	0.415	0.755	0.605	0.450	0.450
TLP-SG	SSU	0.565	0.470	0.460	0.445	0.705	0.605	0.510	0.525
TLP-SG	SSUw	0.585	0.505	0.460	0.415	0.745	0.585	0.485	0.475

*Maximum power in bold*.

The results of the mixed RVs and CVs set-ups are listed in Table 2. Note that, as discussed in Basu and Pan (2011), with mixed RVs and CVs, the SSU test might not perform well. Overall, the SSUw test is the winner. The penalized methods can perform well in some situations, but they do not always outperform the SSUw test. Among the penalized methods, the proposed TLP-S and TLP-SG are competitive against the Lasso and gflasso.

**Table 2:** **Empirical Type I error and Power at the nominal level α = 0.05 based on 200 replicates for the RVs + CVs set-ups with six causal variants and a varying number of non-causal ones**.

**Model fitting**	**Test statistics**	**# of non-causal variants**				**# of non-causal variants**			
		**0**	**8**	**16**	**24**	**0**	**8**	**16**	**24**
		**Null**				**Case 1**			
OLS	*F*-test	0.025	0.045	0.065	0.050	0.760	0.520	0.355	0.385
OLS	Score	0.020	0.045	0.065	0.035	0.760	0.515	0.345	0.350
OLS	SSU	0.060	0.050	0.090	0.030	0.490	0.210	0.125	0.110
OLS	SSUw	0.040	0.035	0.060	0.035	**0.845**	**0.695**	**0.510**	**0.510**
OLS	UminP	0.030	0.055	0.060	0.025	0.715	0.540	0.380	0.410
OLS	Sum	0.055	0.060	0.075	0.045	0.695	0.450	0.315	0.315
OLS	aSum	0.050	0.060	0.065	0.045	0.665	0.435	0.325	0.340
Lasso	1df	0.030	0.045	0.060	0.045	0.750	0.515	0.360	0.375
gflasso_*r = cor*_	1df	0.030	0.030	0.070	0.015	0.760	0.450	0.275	0.415
gflasso_*r* = 1_	1df	0.030	0.030	0.070	0.015	0.765	0.455	0.290	0.385
TLP-S	1df	0.035	0.050	0.050	0.030	0.750	0.540	0.360	0.370
TLP-SG	1df	0.035	0.035	0.065	0.045	0.750	0.515	0.335	0.315
TLP-SG	SSU	0.075	0.060	0.055	0.065	0.495	0.230	0.140	0.105
TLP-SG	SSUw	0.030	0.055	0.055	0.045	**0.845**	0.675	0.435	0.375
		**Case 2**				**Case 3**			
OLS	*F*-test	0.800	**0.765**	**0.720**	0.650	0.655	0.585	0.415	0.375
OLS	Score	0.800	0.755	0.710	0.630	0.645	0.580	0.400	0.360
OLS	SSU	0.275	0.175	0.155	0.160	0.200	0.140	0.110	0.105
OLS	SSUw	0.715	0.705	0.715	**0.665**	0.640	**0.615**	**0.485**	**0.415**
OLS	UminP	0.640	0.615	0.550	0.505	0.530	0.510	0.370	0.345
OLS	Sum	0.190	0.120	0.125	0.100	0.195	0.150	0.090	0.110
OLS	aSum	0.345	0.275	0.270	0.315	0.290	0.225	0.195	0.210
Lasso	1df	**0.805**	0.695	0.640	0.585	0.580	0.555	0.415	0.360
gflasso_*r = cor*_	1df	0.810	0.725	0.625	0.655	0.595	0.570	0.420	**0.415**
gflasso_*r* = 1_	1df	**0.805**	0.725	0.620	0.655	0.590	0.570	0.435	0.395
TLP-S	1df	0.790	0.730	0.680	0.615	0.600	0.570	0.395	0.390
TLP-SG	1df	0.795	0.730	0.620	0.600	0.600	0.555	0.400	0.310
TLP-SG	SSU	0.310	0.185	0.165	0.210	0.205	0.120	0.125	0.120
TLP-SG	SSUw	0.750	0.720	0.650	0.550	**0.675**	0.560	0.460	0.390

*Maximum power in bold*.

An advantage of penalized methods over global tests is the formers' ability for variable selection, narrowing down possible causal variants. We note that causal variant selection is an under-studied problem in genetics, which will become more important when we transition from association studies to causal inference. On the other hand, variable selection via penalized methods or any other methods has yet been fully investigated in the current context with large *n*, small *k*, and more importantly with RVs. In Table 3, we investigate their variable selection performance for one simulation set-up; the results for other set-ups are similar and thus omitted. We show the mean numbers of true positives (TP) and false positives (FP), where a |$\hat{\beta }$_*j*_| > 0.001 is counted as a positive (i.e., non-zero). As expected, the OLS estimates (and the global tests) cannot conduct variable selection with the mean TP and mean FP close to their maximum possible values. Among the penalized methods, a method tends to be either more conservative (fewer FP and fewer TP at the same time) or more liberal (higher FP and higher TP). If we look at the ratio of FP over TP, it seems that the Lasso and TLP-SG are best with the highest ratio, especially for a larger number of non-causal RVs.

**Table 3:** **Mean numbers of TP(sd)/FP(sd) of the methods in Case 2 with both RVs and CVs**.

**Method**	**# of non-causal variants**			
	**0**	**8**	**16**	**24**
OLS	5.9(0.2)/.	5.9(0.3)/7.9(0.4)	5.9(0.3)/15.7(0.5)	6.0(0.2)/23.5(0.7)
Lasso	4.4(1.7)/.	3.7(1.6)/2.9(2.1)	3.5(1.7)/4.7(3.3)	3.2(1.7)/5.9(4.2)
gflasso_*r = cor*_	5.4(1.0)/.	4.8(1.5)/5.1(2.4)	4.1(2.0)/8.0(5.0)	3.5(2.2)/9.3(7.5)
gflasso_*r* = 1_	5.2(1.1)/.	4.5(1.6)/4.8(2.4)	4.3(1.9)/8.9(5.4)	4.1(2.1)/12.3(8.7)
TLP-S	5.4(0.9)/.	4.7(1.1)/4.3(1.5)	4.4(1.1)/7.5(2.1)	4.3(1.1)/11.0(2.9)
TLP-SG	4.7(2.0)/.	4.3(1.8)/4.2(3.2)	3.6(1.6)/5.3(4.5)	3.5(1.5)/6.3(5.0)

*When k = 6, FP is 0 and denoted as “.” after “/”*.

We compare the performance of the parameter estimates in Table 4 for one simulation set-up; the results for other set-ups are similar and thus omitted. As expected, the OLS estimates are almost unbiased, but with the largest mean squared errors (MSEs) due to their large variability. The penalized estimates all have smaller MSEs and larger biases than the OLS estimates. Among the penalized methods, the TLP-S and TLP-SG estimates have much smaller biases, but larger variances and thus larger MSEs than those of Lasso and gflasso. In particular, for a causal effect (β_*c*_), Lasso and gflasso shrink it more toward 0, while TLP-S and TLP-SG give much less biased estimates.

**Table 4:** **Means, sd's and MSEs of some causal (β_*cs*_) and non-causal (β_*ncs*_) variants' regression coefficient estimates when *k* = 30 in Case 2 with both RVs and CVs**.

**Methods**	**β_*cs*_ = 1.5**			**β_*cs*_ = 1.5**			**β_*ncs*_ = 0**		
	**Mean**	**sd**	**MSE**	**Mean**	**sd**	**MSE**	**Mean**	**sd**	**MSE**
OLS	1.59	1.26	3.16	1.54	1.53	4.69	−0.04	1.37	3.77
Lasso	0.93	0.87	1.82	0.84	0.81	1.74	0.01	0.47	0.45
gflasso_*r = cor*_	0.88	0.93	2.11	0.80	0.86	1.96	0.01	0.57	0.64
gflasso_*r* = 1_	0.83	0.92	2.15	0.74	0.86	2.05	0.02	0.55	0.61
TLP-S	1.35	1.14	2.60	1.25	1.15	2.70	−0.03	0.85	1.45
TLP-SG	1.28	1.16	2.72	1.29	1.15	2.70	0.01	0.85	1.44

### 3.2. Mini-exome sequence data

We apply the methods to the mini-exome sequence data from the GAW17 (Almasy et al., 2011). The data set consists of 3205 autosomal genes with 24,487 variants on 697 subjects. The genotypes are obtained from the sequence alignment files provided by the 1000 Genomes Project for the pilot 3 study. The GAW17 data include 200 replicates of three simulated quantitative traits named Q1, Q2, and Q4, where only Q1 and Q2 were influenced by genetic factors. Here we use Q2, which is determined by 72 variants in 13 genes. The true effect sizes of all variants range from 0.2 to 1.2; all variants are positively associated with the trait Q2 but in differential magnitudes.

In this study, we test on each of all causal genes (PLAT, SREBF1, SIRT1, VLDLR, VNN3, PDGFD, BCHE, INSIG1, LPL, RARB, VNN1, and VWF) except GCKR, which contains just one SNP. The number of causal variants (*nC*) in each gene affecting Q2, and some summary statistics of their MAFs and pairwise correlations (COR) are listed in Table 5. Within each gene, most variants are RVs, but a few are CVs with their MAFs larger than 5%. First, we test for any association between Q2 and all variants gene by gene as shown in Table 6, and then test on each gene without its CVs as shown in Table 7.

**Table 5:** **MAFs (%) and pair-wise correlations (COR) in the values of (min, mean, max) for the 12 genes influencing the quantitative trait Q2 in the GAW17 data**.

**Gene**		**All**	**Causal**	**Non-causal**
PLAT	MAF	(0.072,2.098,45.12)	(0.072,0.206,0.574)	(0.072,2.855,45.12)
SREBF1		(0.072,0.699,7.747)	(0.072,0.222,0.43)	(0.072,1.04,7.747)
SIRT1		(0.072,0.858,16.71)	(0.072,0.12,0.215)	(0.072,1.332,16.71)
VLDLR		(0.072,1.047,9.469)	(0.072,0.126,0.287)	(0.072,1.435,9.469)
VNN3		(0.072,4.429,40.53)	(0.072,2.06,9.828)	(0.072,6.501,40.53)
PDGFD		(0.072,4.115,31.56)	(0.072,0.287,0.861)	(0.072,6.303,31.56)
BCHE		(0.072,0.625,14.56)	(0.072,0.105,0.287)	(0.072,1.076,14.56)
INSIG1		(0.072,0.775,3.587)	(0.072,0.072,0.072)	(0.072,1.829,3.587)
LPL		(0.072,1.854,14.490)	(0.072,0.598,1.578)	(0.072,2.076,14.490)
RARB		(0.072,0.352,1.363)	(0.072,0.287,0.502)	(0.072,0.367,1.363)
VNN1		(0.072,2.675,17.070)	(0.574,8.824,17.070)	(0.072,0.215,0.359)
VWF		(0.072,0.944,2.080)	(0.072,0.323,0.574)	(0.359,1.255,2.080)
PLAT	COR	(−0.143,0.002,0.753)	(−0.008,−0.003,−0.001)	(−0.143,0.007,0.753)
SREBF1		(−0.038,0.007,0.635)	(−0.009,−0.004,−0.001)	(−0.038,0.024,0.635)
SIRT1		(−0.044,0.004,0.707)	(−0.004,0.007,0.33)	(−0.044,0.002,0.499)
VLDLR		(−0.135,−0.001,0.331)	(−0.003,−0.002,−0.001)	(−0.135,0.001,0.331)
VNN3		(−0.422,−0.002,0.59)	(−0.104,−0.01,0.072)	(−0.422,−0.001,0.341)
PDGFD		(−0.156,−0.007,0.276)	(−0.007,−0.004,−0.001)	(−0.156,−0.007,0.276)
BCHE		(−0.044,0.001,0.499)	(−0.005,0.004,0.499)	(−0.044,−0.002,0.075)
INSIG1		(−0.010,0.009,0.128)	(−0.001,−0.001,−0.001)	(0.128,0.128,0.128)
LPL		(−0.138,−0.002,0.215)	(−0.010,−0.006,−0.002)	(−0.138,−0.002,0.215)
RARB		(−0.025,−0.003,0.073)	(−0.004,−0.004,−0.004)	(−0.025,−0.005,−0.001)
VNN1		(−0.046,0.038,0.945)	(0.055,0.055,0.055)	(−0.005,0.091,0.945)
VWF		(0.113,0.316,0.564)	(0.265,0.265,0.265)	(0.127,0.246,0.466)

**Table 6:** **Empirical power based on the GAW17 data from 200 replicates of Q2, *k*, and *nC* denote the numbers of the total and causal variants in a gene**.

**Model fitting**	**Test stats**	**Gene(*k*, *nC*)**					
		**PLAT**	**SREBF1**	**SIRT1**	**VLDLR**	**VNN3**	**PDGFD**
		**(28,8)**	**(24,10)**	**(23,9)**	**(27,8)**	**(15,7)**	**(11,4)**
OLS	*F*-test	0.070	0.275	0.360	**0.155**	**0.640**	**0.340**
OLS	Score	0.060	0.260	0.355	**0.155**	**0.640**	0.335
OLS	SSU	0.040	0.025	0.355	0.055	0.185	0.060
OLS	SSUw	0.035	0.245	0.445	**0.155**	0.555	0.320
OLS	UminP	0.065	0.185	0.420	0.120	0.555	0.310
OLS	Sum	0.040	0.075	0.560	0.065	0.410	0.055
OLS	aSum	0.070	0.130	**0.565**	0.095	0.415	0.075
Lasso	1df	**0.100**	0.270	0.285	0.110	0.595	0.300
gflasso_*r = cor*_	1df	0.085	0.195	0.225	0.135	0.555	0.290
gflasso_*r* = 1_	1df	0.085	0.215	0.225	0.135	0.570	0.300
TLP-S	1df	0.065	**0.290**	0.330	0.130	0.630	0.325
TLP-SG	1df	0.025	0.090	0.165	0.075	0.410	0.195
TLP-SG	SSU	0.040	0.015	0.355	0.080	0.220	0.070
TLP-SG	SSUw	0.015	0.085	0.225	0.055	0.330	0.205
		**BCHE**	**INSIG1**	**LPL**	**RARB**	**VNN1**	**VWF**
		**(28,13)**	**(5,3)**	**(20,3)**	**(11,2)**	**(7,2)**	**(6,2)**
OLS	*F*-test	0.375	0.065	**0.305**	0.135	0.750	0.110
OLS	Score	0.365	0.065	0.295	0.135	0.740	0.110
OLS	SSU	0.040	0.090	0.050	0.100	**0.945**	0.170
OLS	SSUw	**0.405**	0.055	0.300	0.130	0.715	**0.210**
OLS	UminP	0.300	0.060	0.285	0.110	0.820	0.170
OLS	Sum	0.180	0.080	0.030	0.145	0.925	**0.210**
OLS	aSum	0.120	0.100	0.090	0.145	0.935	**0.210**
Lasso	1df	0.315	0.050	0.205	0.135	0.655	0.090
gflasso_*r = cor*_	1df	0.300	0.055	0.220	0.120	0.720	0.110
gflasso_*r* = 1_	1df	0.300	0.055	0.215	0.125	0.695	0.110
TLP-S	1df	0.355	0.060	0.270	**0.160**	0.720	0.110
TLP-SG	1df	0.135	0.080	0.115	0.095	0.665	0.080
TLP-SG	SSU	0.045	**0.110**	0.040	0.070	**0.945**	0.140
TLP-SG	SSUw	0.155	0.075	0.135	0.085	0.675	0.145

*Maximum power in bold*.

**Table 7:** **Empirical power based on the GAW17 data without CVs from 200 replicates of Q2, *k*, and *nC* denote the numbers of the total and causal RVs in a gene**.

**Model fitting**	**Test stats**	**Gene(*k*, *nC*)**				
		**PLAT**	**SREBF1**	**SIRT1**	**VLDLR**	**VNN3**
		**(26,8)**	**(23,10)**	**(22,9)**	**(24,8)**	**(12,6)**
OLS	*F*-test	0.070	**0.295**	0.305	0.135	**0.435**
OLS	Score	0.065	**0.295**	0.305	0.135	0.430
OLS	SSU	0.040	0.095	**0.430**	0.075	0.225
OLS	SSUw	0.055	0.270	0.375	0.130	0.390
OLS	UminP	0.060	0.190	0.390	0.125	0.410
OLS	Sum	0.055	0.260	0.350	0.105	0.265
OLS	aSum	0.085	**0.295**	0.380	**0.155**	0.270
Lasso	1df	**0.105**	0.255	0.265	0.140	0.275
gflasso_*r = cor*_	1df	**0.105**	0.195	0.220	0.110	0.255
gflasso_*r* = 1_	1df	0.100	0.225	0.210	0.110	0.280
TLP-S	1df	0.065	0.280	0.295	0.175	0.355
TLP-SG	1df	0.020	0.150	0.215	0.045	0.250
TLP-SG	SSU	0.035	0.090	0.350	0.070	0.265
TLP-SG	SSUw	0.000	0.125	0.235	0.035	0.215
		**PDGFD**	**BCHE**	**INSIG1**	**LPL**	**VNN1**
		**(9,4)**	**(27,13)**	**(4,3)**	**(17,3)**	**(6,1)**
OLS	*F*-test	**0.395**	0.380	0.035	0.340	0.145
OLS	Score	**0.395**	0.380	0.035	0.335	0.145
OLS	SSU	0.200	0.430	0.035	**0.450**	0.195
OLS	SSUw	0.385	0.405	0.035	0.340	0.155
OLS	UminP	0.330	0.305	0.050	0.305	0.115
OLS	Sum	0.155	**0.505**	0.035	0.145	0.035
OLS	aSum	0.195	0.465	**0.075**	0.245	0.135
Lasso	1df	0.315	0.320	0.030	0.230	0.115
gflasso_*r = cor*_	1df	0.310	0.305	0.040	0.255	0.130
gflasso_*r* = 1_	1df	0.335	0.305	0.035	0.260	0.135
TLP-S	1df	0.360	0.345	0.040	0.320	0.115
TLP-SG	1df	0.250	0.430	0.060	0.140	0.110
TLP-SG	SSU	0.175	0.450	0.055	0.440	**0.220**
TLP-SG	SSUw	0.250	0.455	0.055	0.155	0.150

*Maximum power in bold*.

In Table 6, when both RVs and CVs within a gene are included, the identity of the most powerful test differs across the genes: the *F*-test is the winner for the genes VLDLR, VNN3, PDGFD, and LPL; however, for the genes VLDLR, BCHE, VNN1, and VWF, the SSU or SSUw test is the best. The two gflasso-based tests work quite similarly over all genes. The TLP based tests perform best for the genes SREBF1, RARAB, VNN1, and INSIG1. After removing a few CVs in each gene (Table 7), the SSU test recovers good power for the genes PDGFD, BCHE and LPL. The Sum test is the winner for gene BCHE, while the *F*-test based on the OLS estimates perform best for genes VNN3, SREBF1, and PDGFD. For gene VNN1, the TLP-SG with the SSU statistic has the highest power.

A potential advantage of penalized regression is variable selection, which is missing from existing global tests. Table 8 shows the results of causal variant selection by the penalized methods. Overall, each penalized method could eliminate some non-associated variants at the cost of omitting some causal ones. In general, in agreement with simulated data, the Lasso and TLP-SG seem to select fewest variants, including both TPs and FP, while TLP-S and gflasso give higher numbers of both TPs and FPs.

**Table 8:** **Mean numbers of TP(sd)/FP(sd) in the GAW17 data, where *q*1 and *q*0 denote the numbers of the causal and non-causal variants in each gene**.

**Gene(*q*1/*q*0)**	**OLS**	**Lasso**	**gflasso_*r* = *cor*_**	**gflasso_*r* = 1_**	**TLP-S**	**TLP-SG**
	**RVs + CVs**					
PLAT(8/20)	8.0(0.0)/20.0(0.2)	0.8(1.4)/2.1(2.7)	0.6(1.5)/1.9(2.6)	1.5(2.8)/3.8(6.4)	5.0(1.6)/11.4(2.9)	1.7(2.1)/3.9(4.5)
SREBF1(10/14)	10.0(0.0)/14.0(0.1)	2.1(2.6)/2.6(3.0)	2.4(3.1)/3.5(3.9)	6.5(4.4)/8.8(6.1)	6.6(1.6)/8.5(2.4)	2.6(2.1)/3.1(2.2)
SIRT1(9/14)	9.0(0.0)/14.0(0.1)	2.0(1.9)/2.5(2.2)	1.9(2.3)/2.3(3.2)	4.9(3.7)/6.9(5.8)	5.0(1.6)/7.2(2.5)	2.0(1.2)/2.3(1.3)
VLDLR(8/19)	8.0(0.0)/19.0(0.2)	0.8(1.5)/2.7(3.0)	0.8(1.7)/2.6(3.7)	2.1(3.2)/5.4(7.1)	5.0(1.5)/11.7(2.6)	1.5(1.7)/3.8(3.7)
VNN3(7/8)	7.0(0.1)/8.0(0.2)	2.7(1.3)/2.5(1.7)	3.5(1.6)/3.9(2.1)	4.4(2.1)/4.6(2.6)	5.0(1.2)/5.6(1.4)	2.8(1.4)/2.3(1.9)
PDGFD(4/7)	4.0(0.0)/7.0(0.2)	1.2(1.1)/2.3(1.9)	2.1(1.3)/4.2(2.0)	2.5(1.4)/4.4(2.2)	2.9(1.0)/5.3(1.3)	1.2(1.1)/2.0(2.0)
BCHE(13/15)	13.0(0.1)/15.0(0.1)	3.4(3.0)/2.8(2.8)	3.0(3.5)/3.1(3.5)	7.1(5.2)/7.7(6.0)	7.5(2.1)/7.5(2.3)	4.1(3.1)/3.4(3.4)
INSIG1(3/2)	3.0(0.0)/2.0(0.0)	0.2(0.6)/0.4(0.6)	1.0(1.1)/1.1(0.7)	0.8(1.2)/1.1(0.7)	1.6(0.8)/1.4(0.5)	0.7(1.1)/0.7(0.8)
LPL(3/17)	3.0(0.0)/16.9(0.2)	1.0(0.8)/2.9(3.0)	1.1(0.9)/4.0(4.1)	1.4(1.0)/5.4(5.7)	2.5(0.7)/10.8(2.4)	1.2(0.8)/3.2(3.4)
RARB(2/9)	2.0(0.1)/9.0(0.1)	0.7(0.7)/1.2(1.8)	0.8(0.7)/2.1(2.7)	1.0(0.8)/3.2(3.6)	1.6(0.5)/5.1(1.7)	0.8(0.7)/1.4(1.8)
VNN1(2/5)	2.0(0.0)/5.0(0.0)	1.5(0.5)/0.6(1.0)	1.8(0.4)/2.4(1.7)	1.7(0.5)/1.7(1.8)	1.9(0.3)/2.8(1.3)	1.5(0.5)/1.1(1.7)
VWF(2/4)	2.0(0.1)/4.0(0.1)	0.2(0.5)/1.0(1.1)	1.0(0.8)/2.7(1.2)	1.0(0.8)/2.7(1.2)	1.5(0.6)/3.5(0.7)	0.4(0.6)/1.2(1.2)
**RVs only**						
PLAT(8/18)	8.0(0.1)/18.0(0.1)	1.0(1.6)/1.4(2.5)	0.9(1.8)/1.3(2.7)	1.8(2.9)/3.5(6.2)	5.0(1.6)/9.3(2.7)	1.6(1.6)/2.3(2.4)
SREBF1(10/13)	10.0(0.1)/13.0(0.2)	2.1(2.4)/2.2(2.6)	2.3(2.9)/2.9(3.4)	6.7(4.3)/8.5(5.6)	6.5(1.5)/7.4(2.3)	2.7(2.0)/2.7(1.9)
SIRT1(9/13)	9.0(0.0)/13.0(0.1)	2.0(1.9)/2.0(2.4)	1.9(2.3)/2.1(3.0)	4.9(3.7)/6.5(5.6)	5.0(1.6)/6.7(2.1)	2.0(1.5)/2.1(1.8)
VLDLR(8/16)	8.0(0.1)/16.0(0.1)	1.3(1.7)/1.8(2.6)	0.9(1.7)/1.5(3.0)	2.4(3.3)/4.5(6.4)	4.8(1.5)/8.8(2.5)	1.6(1.4)/2.3(2.2)
VNN3(6/6)	6.0(0.0)/6.0(0.1)	1.8(1.3)/1.2(1.3)	2.4(1.5)/2.0(1.8)	2.9(1.9)/2.4(2.1)	3.8(1.2)/3.7(1.3)	1.8(1.2)/1.2(1.3)
PDGFD(4/5)	4.0(0.1)/5.0(0.1)	1.4(1.1)/1.4(1.5)	2.0(1.3)/2.5(1.5)	2.5(1.4)/2.7(1.9)	2.9(0.9)/3.5(1.2)	1.6(1.2)/1.4(1.6)
BCHE(13/14)	13.0(0.1)/14.0(0.1)	3.6(3.1)/2.2(2.7)	3.1(3.6)/2.5(3.4)	8.1(5.0)/8.1(5.6)	7.4(2.0)/6.4(2.2)	3.8(2.3)/2.2(2.1)
INSIG1(3/1)	3.0(0.1)/1.0(0.0)	0.3(0.7)/0.2(0.4)	0.7(1.1)/0.2(0.4)	0.6(1.0)/0.2(0.4)	1.6(0.8)/0.5(0.5)	1.0(1.2)/0.4(0.5)
LPL(3/14)	3.0(0.0)/14.0(0.2)	1.2(0.8)/2.1(2.8)	1.4(1.0)/3.4(4.3)	1.6(1.0)/4.5(5.5)	2.4(0.7)/7.9(2.4)	1.4(0.8)/2.3(2.3)
VNN1(1/5)	1.0(0.0)/5.0(0.1)	0.5(0.5)/0.6(1.0)	0.8(0.4)/2.1(1.8)	0.7(0.4)/1.7(1.9)	0.9(0.2)/2.7(1.3)	0.6(0.5)/1.2(1.8)

## 4. Discussion

In this study we have conducted hypothesis testing to detect the association between a quantitative trait and multiple RVs based on some new penalized regression methods. In addition to the traditional use of penalized regression for variant selection, we have also considered several state-of-the-art grouping pursuit methods that smooth the effect sizes of the variants, either β_*i*_ or |β_*i*_|, in a data-adaptive way, which can be considered as a generalization of the Sum and other genotype pooling/collapsing-based burden tests. In particular, our proposed TLP-SG overcomes several limitations of the Sum and other burden tests. First, by variable selection, the result of TLP-SG is presumably less influenced by the presence of many non-associated variants to be tested. Second, rather than pooling all the variants into a single group or two groups, TLP-SG automatically determines the number of groups to be formed based on the given data. Furthermore, since TLP-SG shrinks the effects sizes |β_*i*_|, not β_*i*_, toward each other, it is robust to varying association directions of the causal variants. However, based on our studies on both simulated and real sequence data, we found that TLP-SG and other penalized methods sometimes might be more powerful than some existing global tests, though they do not always outperform the SSU or SSUw test. The discovery of no uniform gain of penalized methods over existing global tests is interesting and even surprising, and can be due to non-optimal implementation of the penalized methods in several aspects. First, the selection of the tuning parameters based on the model selection criterion AIC may not be optimal. As an example, in a simulated dataset, when we set the tuning parameters to properly group the variants, the estimates were quite close to the true values, but the corresponding AIC was less desirable, leading to choosing other low performing tuning parameters. Importantly, there is no theory yet to justify the applicability of AIC for the gflasso- and TLP-based methods; in particular, it is unclear how to count the effective number of parameters in the AIC. Alternatively, one may want to try a more popular model selection method, multi-fold cross-validation. However, for RVs as considered here, if we divide the data into multiple folds, the training data may contain several monomorphic variants, causing non-identifiability of their corresponding effect sizes. Second, due to the repeated model-fitting with many permuted datasets, to save computing time, we only searched relatively few grid points for the tuning parameters, which might not have covered some suitable tuning parameter values. These are all issues to be addressed in the future.

Another non-convex penalty is SCAD (Fan and Li, 2001), which as TLP aims to reduce the biases of large coefficient estimates resulting from the Lasso or *L*_1_ penalty. Although SCAD can be equally applied and compared here, we chose the TLP as a representative of non-convex penalties for its good properties: as shown by Shen et al. (2012), *L*_0_ regularization is optimal in variable selection, and its computational surrogate, TLP, shares the same property for sufficiently small tau; furthermore, the variable selection consistency of TLP regularization also led to enhanced parameter estimation and prediction in numerical studies with finite sample sizes. Nevertheless, we note that, penalized regression methods have been intensively studied for high-dimensional data, but not for the type of data considered here, which are low dimensional but with RVs as sparse predictors.

In summary, the established benefit of penalized regression for variable selection and risk prediction for high-dimensional data (Kooperberg et al., 2010) did not seem to directly translate into substantial power gains in genetic association testing. In addition to the current work, there exist three recent reports (Croiseau and Cordell, 2009; Martinez et al., 2010; Basu et al., 2011) questioning the effectiveness of the Lasso penalized regression in hypothesis testing, while Basu et al. (2011) showed that several variable selection approaches did not outperform some global tests (e.g., the SSU or SSUw test) for association analysis of CVs. Due to the limitations mentioned above, we cannot conclude here that any penalized regression method would not outperform exiting global association tests; rather, further investigation on enhanced tuning parameter selection and better choice of the test statistic is warranted. Finally, we note that the capability of variable selection by penalized regression can be useful, e.g., in narrowing down causal variants.

### Conflict of interest statement

The authors declare that the research was conducted in the absence of any commercial or financial relationships that could be construed as a potential conflict of interest.
